# New insights in animal models of neurotoxicity-induced neurodegeneration

**DOI:** 10.3389/fnins.2023.1248727

**Published:** 2024-01-08

**Authors:** Coral Sanfeliu, Clara Bartra, Cristina Suñol, Eduard Rodríguez-Farré

**Affiliations:** ^1^Institut d’Investigacions Biomèdiques de Barcelona (IIBB), Consejo Superior de Investigaciones Científicas (CSIC), and Institut d’Investigacions Biomèdiques August Pi i Sunyer (IDIBAPS), Barcelona, Spain; ^2^PhD Program in Biotechnology, Facultat de Farmàcia i Ciències de l’Alimentació, Universitat de Barcelona, Barcelona, Spain

**Keywords:** neurodegeneration, experimental models, neurotoxic agents, neurotransmission, neuroinflammation, Parkinson’s disease, Alzheimer’s disease

## Abstract

The high prevalence of neurodegenerative diseases is an unintended consequence of the high longevity of the population, together with the lack of effective preventive and therapeutic options. There is great pressure on preclinical research, and both old and new models of neurodegenerative diseases are required to increase the pipeline of new drugs for clinical testing. We review here the main models of neurotoxicity-based animal models leading to central neurodegeneration. Our main focus was on studying how changes in neurotransmission and neuroinflammation, mainly in rodent models, contribute to harmful processes linked to neurodegeneration. The majority of the models currently in use mimic Parkinson’s disease (PD) and Alzheimer’s disease (AD), which are the most common neurodegenerative conditions in older adults. AD is the most common age-related dementia, whereas PD is the most common movement disorder with also cases of dementia. Several natural toxins and xenobiotic agents induce dopaminergic neurodegeneration and can reproduce neuropathological traits of PD. The literature analysis of MPTP, 6-OH-dopamine, and rotenone models suggested the latter as a useful model when specific doses of rotenone were administrated systemically to C57BL/6 mice. Cholinergic neurodegeneration is mainly modelled with the toxin scopolamine, which is a useful rodent model for the screening of protective drugs against cognitive decline and AD. Several agents have been used to model neuroinflammation-based neurodegeneration and dementia in AD, including lipopolysaccharide (LPS), streptozotocin, and monomeric C-reactive protein. The bacterial agent LPS makes a useful rodent model for testing anti-inflammatory therapies to halt the development and severity of AD. However, neurotoxin models might be more useful than genetic models for drug discovery in PD but that is not the case in AD where they cannot beat the new developments in transgenic mouse models. Overall, we should work using all available models, either *in vivo*, *in vitro*, or *in silico*, considering the seriousness of the moment and urgency of developing effective drugs.

## 1 Introduction

A wide spectrum of disease animal models has been used in preclinical research of neurodegenerative diseases for decades. However, all too often positive results with new drugs are not replicated in the clinical setting, thus contributing to raise opposition against animal experimentation. Therefore, it is time to reassess the models we use and look for experimental models of neurodegeneration that are more faithful to human neuropathological processes. At present, genetic models are widely used. These models refer to an organism, often a laboratory animal such as a mouse, that has been genetically modified to carry specific genes or mutations associated with a human trait, disease, or condition. However, pathologies driven by specific mutations would only mimic a minority of human neurodegenerative processes. That problem is exacerbated when we consider age-related diseases that make up the bulk of neurodegenerative cases in our increasingly old population (Azam et al., 2021). An option is to use classic and new neurotoxicity-based animal models that mimic external and internal environmental insults received throughout lifetime but mainly during the aging process. These models rely on the functional or structural alterations of the central nervous system (CNS) induced by natural toxins and xenobiotics that ultimately cause neurodegeneration (Shaw and Höglinger, 2008).

Small rodents, such as mice and rats, can be treated with specific neurotoxic agents to induce neurodegeneration and test the neuroprotection of drugs, allowing comprehensive studies from the molecular level to complex behavior. Although other rodents or primates are also suggested for specific purposes, such as gerbils for ischemic neurodegeneration or aged primates for AD (Shin et al., 2019; Trouche et al., 2023), the advantages of mice and rats are undeniable. These advantages include similarities in brain anatomy, short lifespan, rapid breeding, small size, cost-effectiveness and ease of maintenance, behavioral testing capabilities, genetic manipulation, and disease modeling (Bryda, 2013). Furthermore, animal experimentation remains essential in the preclinical studies of many diseases, as *in silico*, *ex vivo*, and *in vitro* testing cannot reproduce the complexity of the body–brain interaction and the neuropsychological processes. It should be added the need to address differential sex neurodegenerative processes (Bianco et al., 2023) and responses to drugs (Buoncervello et al., 2017) that can be fulfilled by using male and female rodents.

Here, we critically analyze known and new neurotoxic agent-mediated models of neurodegeneration as reliable models that may complement gene-mediated approaches and contribute to demonstrate druggable targets in neurodegenerative disease. We focus on rodent models of neurotoxic agents that cause pathological changes in neurotransmission and neuroinflammation mechanisms. We propose these alterations as relevant early factors in the progressive loss of structure or function of neurons ultimately leading to cognitive loss and dementia in neurodegenerative diseases. Several models of neurotoxicity-induced cholinergic alteration or neuroinflammation are used to test new drugs for Alzheimer’s disease (AD), the most common age-related dementia. CNS neurodegeneration can lead to motor diseases such as Parkinson’s disease (PD), the most common movement disorder that also shows an increased risk of dementia. Specifically, neurotoxin models of PD are successful in reproducing the main aspects of the disease, and we address dopaminergic neurodegeneration models more broadly.

## 2 Validated and new experimental models of neurodegeneration

### 2.1 Models based on dopaminergic neurodegeneration

In the CNS, there exist four main dopaminergic systems: nigrostriatal and ventral tegmental, mesocortical, mesolimbic, and tuberoinfundibular area. The progressive, lengthy, relatively selective, extensive, and irreversible loss of dopaminergic neurons in the substantia nigra (SN) pars compacta and their projections in the corpus striatum (caudate nucleus and putamen) constitute the main pathological manifestation of the neurodegenerative disorder present in PD. A second hallmark of PD is the presence in the nigrostriatal dopaminergic neurons of characteristic cytoplasmic inclusions known as Lewy bodies, which contain fibrils of misfolded α-synuclein protein. The clinical features of the striatal loss of dopamine in PD are resting tremor, postural and gait dysfunction, bradykinesia, muscular rigidity, etc. (Braak and Del Tredici, 2008). In addition, PD encompasses in its evolution non-motor manifestations (e.g., depression, sleep alteration, declining memory, and psychoses) which involve the other dopaminergic systems and CNS structures.

PD is expressed in two forms: familial, with approximately 5% of cases, and sporadic or idiopathic with 95%. Sporadic PD represents the second most frequent neurodegenerative disease, only after AD, affecting approximately 1% of the elderly population (Ray Dorsey et al., 2018; Ou et al., 2021), thus implying a high burden for affected people and for public health. Epidemiological studies have reported an association between exposure to environmental chemicals, particularly pesticides, and increased risk of PD (Kuopio et al., 1999; Pedro-Cuesta Jd et al., 2009; Goldman, 2014).

In recent years, the above classical definition of PD is becoming to be considered a systemic or multicentric and staging pathology, involving the entire nervous system, i.e., the CNS, the peripheral (PNS), and the enteric nervous system (ENS) (Braak and Del Tredici, 2008; Braak and Del Tredici, 2017). In this concept, PD is viewed as a gut–brain neurodegenerative process in which the central pathology is an α-synucleinopathy (Natale et al., 2010; McCann et al., 2014; Li et al., 2023).

**Figure 1:**
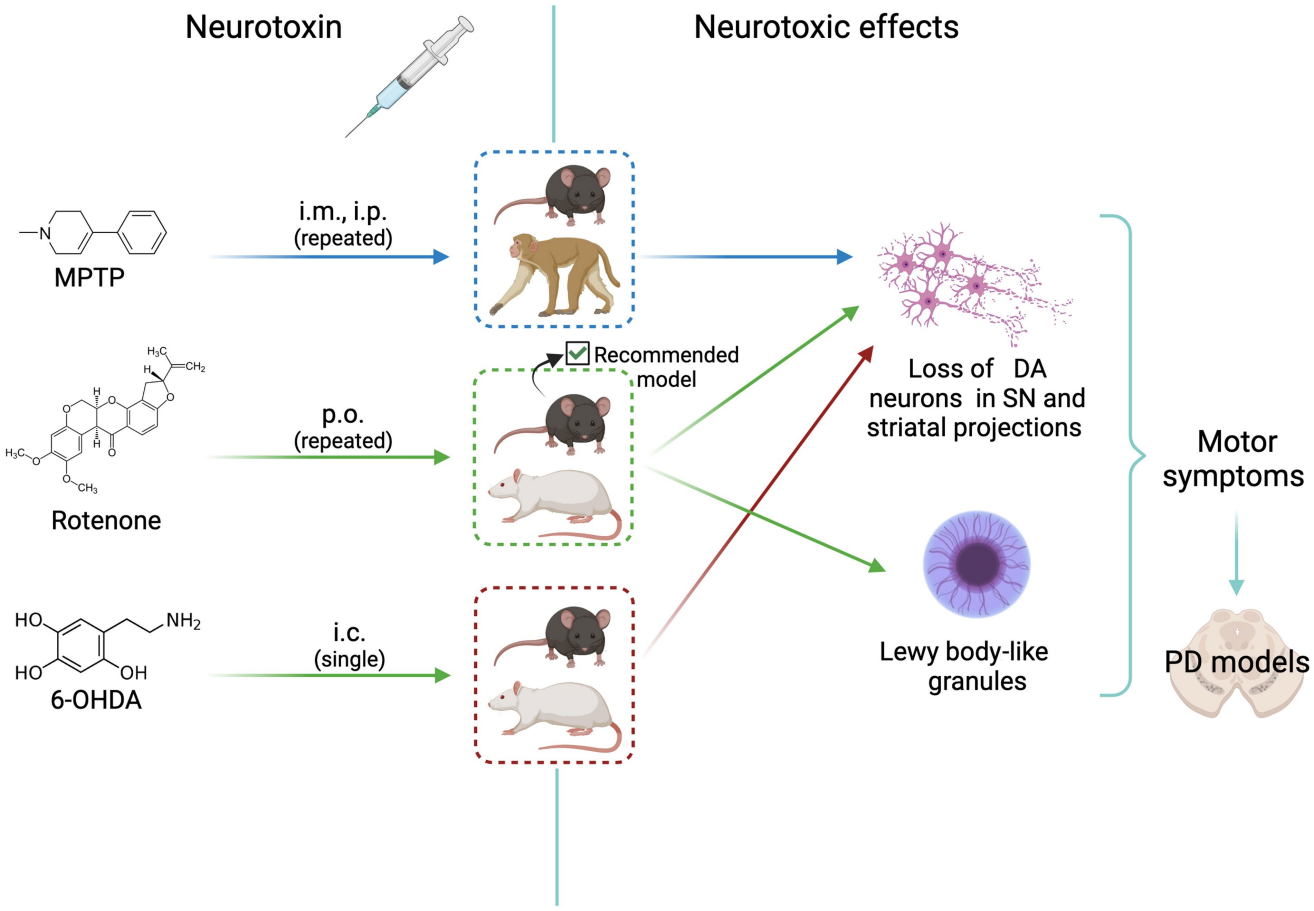
Scheme of animal models of neurotoxicity-induced dopaminergic neurodegeneration. MPTP, rotenone, and 6-OHDA induce neurotoxic effects that mimic Parkinson’s disease signs. All agents cause loss of dopaminergic neurons. The most complete and reliable model is that induced by rotenone treatment of C57BL/6J mice, that shows the formation of Lewy body-like granules. See text for details. i.m., intramuscular; i.p. intraperitoneal; p.o. *per os* (oral); i.c., intracerebral (medial forebrain bundle or striatum); DA, dopaminergic; MPTP, 1-methyl-4-phenyl-1,2,3,6-tetrahydropyridine; PD, Parkinson’s disease. Created with BioRender.com.

The goal of experimental models for sporadic PD is to reproduce as close as possible the pathological and clinical manifestations of human disease (there are no spontaneous PD observed in animals). Three models are currently used to investigate the pathophysiology and the pharmacology of PD. Figure 1 shows the scheme of the treatment route, the animal species used, and the neurotoxic effects elicited in these models, as detailed below.

#### 2.1.1 1-methyl-4-phenyl-1,2,3,6-tetrahydropyridine model of PD

Forty years ago, Langston et al. (1983) reported that several young adults in California developed a marked and irreversible parkinsonism shortly after using intravenously a new synthetic illicit drug (Nonnekes et al., 2018). This heroin-like drug resulted to become meperidine, which contained 1-methyl-4-phenyl-1,2,3,6-tetrahydropyridine (MPTP) as a contaminant. The toxicity of MPTP in non-human primates (NHP) displayed clinical signs and nigrostriatal neuropathology similar to the human sporadic PD, henceforth providing an experimental tool for investigating parkinsonism (Burns et al., 1983; Langston et al., 1983).

MPTP as a model of xenobiotic-induced parkinsonism has been studied extensively for many years and constituted the gold standard for experimental PD research. It appeared as a selective neurotoxic xenobiotic inducing dopaminergic nigrostriatal neurodegeneration. As a lipophilic compound, MPTP crosses easily the blood–brain-barrier (BBB) to reach the CNS but it has no toxicity. There it is taken up by astrocytes where MPTP is swiftly oxidized in two steps by monoamine oxidase B (MAO-B) to the neuroactive metabolite 1-methyl-4-phenylpyridinium (MPP+). Pharmacological inhibition of MAO-B protects against MPTP toxicity, while MPP+ systemic administration does not cross the BBB and is only neurotoxic by intracerebral injection. MPP+ released from astrocytes is captured into dopaminergic neurons by the dopamine transporter (DAT) located in the neuronal membrane. This dopamine carrier has a greater expression in neurons of the SN than in other areas and shows a high affinity for MPP+. The final step of MPP+ neurotoxicity consists of the inhibition of complex I activity in mitochondria, resulting in dopamine oxidation to form quinones and reactive oxygen species (ROS) which cause cell death. Due to the high expression of DAT and its high affinity for MPP+ neuronal death is nearly circumscribed to SN and ventral tegmental area, a neuropathology mimicking that of PD (Blesa and Przedborski, 2014).

Different animal species have been and are used to model PD by means of MPTP. Despite its limited use (cost, adequate facilities, technical skills, and ethical issues), NHPs are considered for their close physiology and brain structure with humans the best standard for studies of MPTP-induced parkinsonism (Emborg, 2017; Masilamoni and Smith, 2018; Khan et al., 2023). The NHP model provides the various species of MPTP-treated monkey’s motor, behavioral, and neuropathological features observed in PD patients. However, there are divergences in certain pathophysiological pictures described. The different regimes of treatment may account for the diverse results observed. Thus, NHP treated with acute high doses of MPTP (e.g., 1–4 mg/kg/day, i.p., for 5 days) develop in a short time acute and long-term effects with signs of motor parkinsonism and a clear nigrostriatal loss of dopaminergic neurons and their synaptic terminals in striatum (Waters et al., 1987; Masilamoni and Smith, 2018). In front of this classical treatment, it has been observed that chronic treatment with low doses of MPTP (e.g., 0.2–0.5 mg/kg i.m. once a week) after approximately 21 weeks, and the NHP displayed motor signs non-motor manifestations of PD (Masilamoni et al., 2011). In this chronic study, besides the important nigrostriatal dopaminergic loss, MPTP also caused the loss of non-nigrostriatal dopaminergic neurons and noradrenergic neurons in the locus coeruleus (Oertel et al., 2019), a characteristic pathological feature observed in PD patients. In other experiments, a NHP loss of serotonergic neurons in the dorsal raphe has also been observed (Duperrier et al., 2022).

When comparing the features of MPTP-induced parkinsonism with the neuropathology of patients, the most striking difference is the absence of Lewy bodies and Lewy neurites in the model animals, be NHP or mice.

The most widely used animal model for MPTP-induced parkinsonism is the pigmented C57BL/6 mice. This is the rodent model of choice because rats do not respond to MPTP toxicity and different strains of mice have variable and erratic effects to its exposure (Tello et al., 2022; Khan et al., 2023). The neurotoxic action of MPTP on C57BL/6 mice follows the same pattern as described above for NHP. Here again, the rodent model shows certain variability according to the dose level and the time span of treatment (Merghani et al., 2021; Khan et al., 2023). In this way, mice MPTP-treated with 30 mg/kg/day i.p. for 6 days (a dose much greater than those used with NHP) developed degeneration of the nigrostriatal pathway in both the SN and the striatum, as shown by loss of dopaminergic neurons immunostained for tyrosine hydroxylase (TH), depletion of striatal dopamine, activation of glial cells in the nigrostriatal pathway, and impairment in behavioral tests (Bhurtel et al., 2019). It has been proposed that oxidation of dopamine plays a role in the loss of dopaminergic neurons in SN. However, pharmacological depletion of dopamine does not protect against nigrostriatal degeneration in MPTP-treated C57BL/6 mice (Hasbani et al., 2005).

In humans, sex differences have been reported for PD, especially a higher incidence in men than in women (Miller and Cronin-Golomb, 2010). The MPTP model in NHP has been carried out in female (Waters et al., 1987; Masilamoni et al., 2011) and male (Burns et al., 1983; Masilamoni and Smith, 2018), but to the best of our knowledge, no sex differences were studied. Most research using C57BL/6 mice includes only male subjects; nevertheless, comparison of male and female mice treated with small, chronic doses of MPTP revealed selective motor impairment only in male subjects (Antzoulatos et al., 2010).

#### 2.1.2 The rotenone model of PD

Rotenone is a toxic isoflavone present in certain plants of the genera *Derris* and *Lonchocarpus*. It has been used as a broad-spectrum ichthyotoxin, insecticide, and pesticide. The epidemiological observation that chronic exposure to systemic pesticides such as rotenone is associated with an increased risk of PD led to investigate whether rotenone produces in experimental animal manifestations of PD (Betarbet et al., 2000). The positive results of that research established the rotenone model, since then, one of the most employed in PD studies.

Rotenone as a lipophilic compound crosses the biological membranes, including the BBB and, in contrast with MPTP, acts directly in the cell inhibiting the electron transport chain of the mitochondrial complex I (NADH: ubiquinone oxidoreductase). This effect reduces ATP formation and can produce ROS and lead to cell death. In contrast with MPTP, there is no selective toxicity. Rotenone acts as a toxin on all cells of the organism, and the CNS manifestations are related to the greater vulnerability of certain systems. The chronic inhibition of mitochondrial complex I by rotenone determines a highly selective nigrostriatal dopaminergic degeneration associated with motor signs (hypokinesia and rigidity). A specific mark of the rotenone-treated rats is that nigral neurons accumulate fibrillary cytoplasmic inclusions containing α-synuclein and ubiquitin, morphologically similar to Lewy bodies (Johnson and Bobrovskaya, 2015).

The original rotenone model, briefly seen above, established an optimal dose for producing the PD pathology: 2–3 mg/kg/day administered by osmotic pump in Lewis rats for periods of 7 days to 5 weeks of continuous administration. Motor and behavioral tests, biochemistry, and neuropathology defined the model. Nonetheless, a great number of variants in animal species, dosage, via and span of administration, and methods of evaluation have appeared over time. Numerous articles testing drugs or biologicals use a quick behavioral or motor test in a short-term, high-dose pseudorotenone model. It is well known that high doses of rotenone, up to 100 mg/kg, for a short period produced systemic toxicity and unspecific lesions in the brain such as necrosis in the striatum. The ability of the different rotenone model variants to reproduce the clinical features of PD has been analyzed in various reports (Johnson and Bobrovskaya, 2015; Tello et al., 2022; Khan et al., 2023).

A question concerning the usefulness of the MPTP and rotenone models in inducing the clinical neurological signs and neuropathology of sporadic PD is still open. Two recent studies have addressed the issue by comparing experimentally both models. Bhurtel et al. (2019) treated groups of four C57BL/6 mice with MPTP 30 mg/kg/day i.p. or with rotenone 2.5 mg/kg/day i.p. for 6 days and compared the neurotoxicity of both compounds. A battery of behavioral tests, neurochemical analysis, and immunohistochemical staining were applied to both treated groups. It was determined that MPTP induces dopaminergic nigrostriatal lesions with neuronal loss and behavioral impairment as it has been seen above, but not rotenone (at least at the dosing level administered). The rotenone group only displayed neurodegeneration and glial activation in the hippocampus. The authors propose that MPTP is more specific than rotenone to produce dopaminergic neurodegeneration.

In contrast, in a comprehensive study, Zhang et al. (2022) dosed groups of 30 C57BL/6 mice with MPTP 20 mg/kg i.p. twice a week over 6 consecutive weeks, or with rotenone 30 mg/kg by gavage following the same schedule. To compare the effects of both neurotoxic agents, neurobehavior, neuropathology, neurochemistry, and mitochondrial function were assessed in treated and control groups by means of a large and complete battery of adequate techniques. Locomotor activity and coordination and exploratory behavior were significantly lower in both treated groups compared with controls. The MPTP-treated group showed the typical loss of dopaminergic neurons in SN pars compacta and the reduction of TH content in SN and striatum that were more significant than in the rotenone group. Remarkably, oxygen consumption in mitochondrial complex I in the SN was significantly reduced in the rotenone group compared with the MPTP group. As expected, Lewy bodies were only present in SN neurons in the rotenone group.

The striking difference in the rotenone effects observed in these studies may belong to the low dose of rotenone, via administration used, and duration of treatment. After pondering the different procedures described in the literature for the rotenone model, it appears as more reproducible and consistent those described by Betarbet et al. (2000) and Zhang et al. (2022).

The rotenone model has also provided an experimental approach to the hypothesis considering sporadic PD as a staging disease that can start in the ENS and progress through the nervous system until reaching the brain (Braak and Del Tredici, 2008; Braak and Del Tredici, 2017). In relation to that concept, the administration of rotenone intragastrically to C57BL/6J mice 5 mg/kg 5 days a week for 1.5–3 months induced α-synuclein accumulation in all the nervous system structures considered for the progression of PD. Namely, it starts in the ENS and follows the dorsal motor nucleus of the vagus, the intermediolateral nucleus of the spinal cord and the SN. α-synuclein phosphorylation in the ENS and other nervous structures was also observed. The alterations are sequential appearing only in synaptically connected structures. The rotenone model, then, may be apt for studying the α-synucleinopathies (Pan-Montojo et al., 2010).

The rotenone model has been commonly studied in male rats and mice. Related to sex, one study reported that rotenone-treated male rats show a decrease in testosterone levels in blood plasma (Alam and Schmidt, 2004). This decrease is one of the comorbidity signs found in male PD patients (Okun et al., 2002) and is probably linked to the more severe motor deficits in male rodents (Antzoulatos et al., 2010). Furthermore, sex differences have been shown in female rats that display a decreased sensitivity to rotenone-induced neurodegeneration (De Miranda et al., 2019). These female subjects showed less inflammation and less accumulation of α-synuclein than matched males. Sex differences in sensitivity may reflect the higher male ratio in human PD.

#### 2.1.3 6-hydroxydopamine model of PD

6-Hydroxydopamine (6-OHDA) is a neurotoxic xenobiotic analogue to the catecholaminergic transmitters. Its systemic administration produces a long-lasting depletion of noradrenaline and dopamine in peripheral nerve terminals but not in the CNS as it does not traverse the BBB. Injecting intracerebrally 6-OHDA in experimental animals is the oldest method of modelling PD (Ungerstedt, 1968). Although with many refinements added over time, 6-OHDA continues to be used to investigate the pathophysiology of PD as well as the preclinical aspects of putative neuroprotective or therapeutic drugs (Simola et al., 2007; Hernandez-Baltazar et al., 2017). By means of stereotaxic surgery, 6-OHDA is injected into the medial forebrain bundle that destroys the dopamine neurons projecting from the midbrain to the striatum. To increase the selectivity of 6-OHDA for dopaminergic neurons, animals must be treated previously with a blocker of the noradrenaline transporter, usually with desipramine. Stereotaxic administration of 6-OHDA is also done in the striatum, causing a more progressive and less severe lesion. Most studies on the motor deficits of PD use the ipsilateral injection of 6-OHDA in the right medial forebrain bundle or in the right mid-striatum to obtain the model of contralateral rotational behavior. This model works satisfactorily in many species (monkeys, rats, and mice). An application of interest is the use of the mouse model of rotation for tissue transplantation in the damaged zone, an area of stem cell research (Bagga et al., 2015; Guimarães et al., 2021). The 6-OHDA model in its rotational version is also employed in transgenic mice to search for very specific aspects of dyskinesias (Castela et al., 2023).

The mechanisms of neurotoxicity and usefulness of the 6-OHDA model are to a great extent similar to those of MPTP although in several aspects 6-OHDA can provide more precise and detailed neuropathological information. Both models do not express α-synuclein, but in a recent study (Cui et al., 2023), 6-OHDA injected in the rat medial forebrain bundle did not induce expression of α-synuclein in the brain, but at 5 weeks, post-lesion were detected as having increased levels of phosphorylated α-synuclein in ileum and colon. That finding may be related to the multistage theory seen above on the axis gut–brain for sporadic PD.

Male rats and mice are the customary animals used in the 6-OHDA model. A study of bilateral partial lesions with 6-OHDA in rats of both sexes observed a reduction of locomotor activity in aged male subjects but not in female subjects (Cass et al., 2005). Moreover, it has been shown that the effects of 6-OHDA striatal lesions on spatial cognition are sex-specific in rats and testosterone-dependent in male subjects (Betancourt et al., 2017).

### 2.2 Models based on cholinergic neurodegeneration

**Figure 2:**
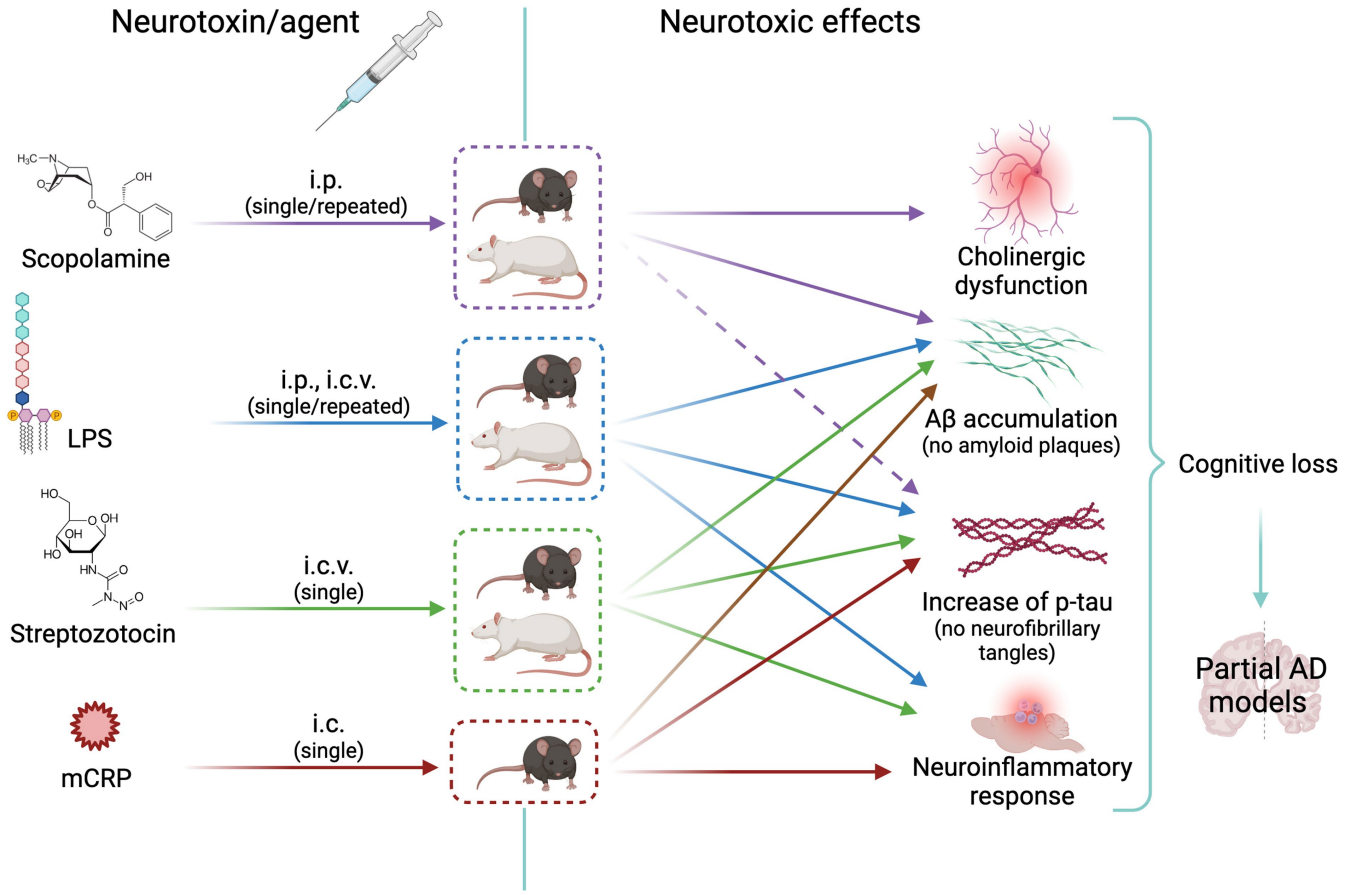
Scheme of animal models of neurotoxicity-induced cholinergic or neuroinflammatory changes leading to Alzheimer’s disease-like neurodegeneration. The cholinergic agent scopolamine is widely used as inducer of memory loss; it increases tau synthesis without increasing p-tau (effect represented by a dashed arrow) or Aß. LPS, streptozotocin and mCRP induce a neuroinflammatory response and mild amyloid and tau pathologies with increase of p-tau and Aß. See text for details. i.p. intraperitoneal; i.c.v., intracerebroventricular; i.c., intracerebral (hippocampus); AD, Alzheimer’s disease; Aβ, Amyloid beta; LPS, lipopolysaccharide; mCRP, monomeric C reactive protein. Created with BioRender.com.

Cholinergic neurotransmission plays an important role in encoding new memories and other high brain functions. This fact together with the cholinergic atrophy paralleling cognitive decline in aging and AD led to the early cholinergic hypothesis of AD. It is known that neurofibrillary degeneration and death of forebrain cholinergic neurons cause presynaptic cholinergic denervation in AD (Hampel et al., 2018). Cholinergic neurotransmission enhancers acting through acetylcholinesterase (AChE) inhibition (donepezil, rivastigmine, and galantamine) have been used for decades to temporarily slow AD progression. AChE is increased around amyloid plaques and neurofibrillary tangles, the two main neuropathological hallmarks in the AD brain, suggesting a vicious cycle between AChE and amyloid and tau dysregulation (García-Ayllón et al., 2011). Preclinical and clinical research of new drugs moved soon to anti-amyloid and anti-tau therapies according to the amyloid and tau hypotheses of AD [see the history of AD hypotheses in Liu et al. (2019)]. It is estimated that less than 1% of cases are caused by autosomal dominant mutations and the majority is the sporadic form of AD. The prevalence of this devastating disease is approximately 10% in the population aged 65 years and older (Alzheimer’s Association Report, 2022; Manly et al., 2022). However, only recent developments in anti-amyloid immunotherapy opened the path to disease-modifying drugs with current hope in the monoclonal antibody lecanemab (Van et al., 2023) and others in development. Rat and mouse models of cholinergic neurodegeneration are still widely used for screening pro-cognitive and anti-dementia drugs, including those addressed to AD. The first row of Figure 2 shows the scheme of the treatment route, the animal species used, and the neurotoxic effects elicited with scopolamine, as detailed below.

#### 2.2.1 Scopolamine model of AD

Scopolamine is a tropane alkaloid found in several plants. It is a cholinergic neurotoxin due to its properties as a competitive antagonist of the muscarinic receptors. At the right dose, it is used as anti-emetic drug. Scopolamine is lipophilic and easily crosses the BBB causing a powerful effect in the CNS. Single or repeated i.p. administration of scopolamine in mice and rats induces cholinergic dysfunction and increase of AChE that further cleaves acetylcholine at the synapses leading to amnesia and cognitive loss. Doses administered range from 0.4 mg/kg to 3 mg/kg i.p., with longer-lasting memory impairment after repeated daily administration for one or more weeks than a single injection (Rahimzadegan and Soodi, 2018; Chen and Yeong, 2020; Kose et al., 2023). This model is mainly used for behavioral studies, but some authors report that scopolamine induces an imbalance of the amyloid-beta precursor protein (APP) processing to the amyloidogenic pathway causing intraneuronal accumulation of amyloid-beta (Aß) and also increased tau synthesis (Chen and Yeong, 2020; Kose et al., 2023). Noticeably, the scopolamine model in mice and, to a minor extent, in rats, was found the most widely used *in vivo* model for multitarget anti-AD drug discovery in a bibliometric analysis of the last 3 decades (Sampietro et al., 2022). Male and female rats experience a similar increase in AChE and memory loss in response to scopolamine (Ali and Arafa, 2011) although sex differences in fine cholinergic responses cannot be discarded.

### 2.3 Models based on neuroinflammation-induced neurodegeneration

Neuroinflammation, that is, the inflammatory response within the CNS, is a double-edged sword depending on its triggering cause, its duration until it resolves, and the stage of frailty of the organism (Disabato et al., 2016). The innate immune cells of the nervous system will fight against infectious agents or local injuries, but if their response is deregulated, they can contribute to further injury and either initiate or worsen neurodegeneration. This is the case of the pathological reactive phenotype of microglia, the first-line innate immune brain cells in AD and other neurodegenerative diseases (Hammond et al., 2019). Systemic inflammation may also contribute to brain neurodegeneration (Chouhan et al., 2022). That interaction is highly relevant in old age when sustained low-grade inflammation in several organs and tissues is a key driver of age-related diseases including neurodegenerative diseases (McGeer and McGeer, 2004). Therefore, drug protection against neuroinflammation will halt brain inflammation and decrease the risk of late-onset neurodegeneration.

The relevance of neuroinflammatory mechanisms on the triggering and progression of AD promoted the use of various models of neuroinflammation in AD. The second, third, and fourth rows of Figure 2 show the scheme of the treatment route, the animal species used, and the neurotoxic effects in the three selected AD models described below.

Mixed proinflammatory and dopaminergic toxin models were developed for PD. Other neurodegenerative disease such as Huntington’s disease or amyotrophic lateral sclerosis also show neuroinflammation, but this is not an early mechanism and specific animal models of neuroinflammation were not established (Vinther-Jensen et al., 2016; Chakraborty and Diwan, 2022).

#### 2.3.1 Lipopolysaccharide model of AD

Lipopolysaccharide (LPS), the major component of the outer membrane of Gram-negative bacteria, is widely used to induce neuroinflammation in rats and mice. There are a variety of protocols using different LPS serotypes and doses ranging from 0.3 mg/kg to 10 mg/kg i.p. and 0.25 μg to 50 μg i.c.v., with single or repeated administration over one or more days (Nava Catorce and Gevorkian, 2016; Zakaria et al., 2017). The systemic treatment through i.p. injection in mice is very convenient for testing protective anti-inflammatory agents (Nava Catorce and Gevorkian, 2016). The sickness behavior response of mice to LPS peripheral infection parallels the release of proinflammatory cytokines in the brain and blood (Molina-Martínez et al., 2021). The mechanisms of microglia activation by LPS have been fully characterized either after peripheral (i.p.) or central (i.c.v.) administration in mice and rats. LPS elicits a robust neuroinflammatory response followed by cognitive loss and neurodegeneration although the response and the brain areas affected may differ according to the administration regimen and the age of the animal (Nazem et al., 2015; Zakaria et al., 2017; Decandia et al., 2023). Furthermore, some clinical evidence supports an involvement of LPS from Gram-negative bacteria resident in the gastrointestinal tract as a potential contributor to the onset of AD (Zhao et al., 2017). Rodents dosed peripherally with LPS make a reliable model of AD neuroinflammation. In addition, LPS may promote increased brain Aβ levels and tau pathology although the wide diversity in the treatment design and the LPS serotypes used do not yield clear conclusions (Batista et al., 2019). Male and female rodents are used with good results (Nava Catorce and Gevorkian, 2016). However, there are sex differences in the pattern of immune responses to LPS treatment that should be taken into account when evaluating proinflammatory biomarkers (Sharma et al., 2018).

#### 2.3.2 Streptozotocin model of AD

Streptozotocin (STZ) is a naturally occurring glucosamine-nitrosourea compound identified in a Streptomyces bacteria strain. The first use of this toxin is to model diabetes in rodents as peripheral injection of STZ damages pancreatic β-cells. Impaired insulin sensitivity is indeed a risk factor for AD development. In the brain, both i.p. and i.c.v. injections of STZ cause neuroinflammation and cognitive deficits in mice and rats (Nazem et al., 2015). Interestingly, bilateral i.c.v. injections of STZ in mice elicit chronic neuroinflammation and increased levels of Aβ and hyperphosphorylated tau (p-tau) in the hippocampus (Fan et al., 2022). Therefore, centrally administered STZ, at doses 1 or 3 mg/Kg i.c.v., is considered a better model of sporadic AD than peripherally administered STZ (Nazem et al., 2015; Fan et al., 2022). A sex-difference study reported that female rats respond less than male subjects to i.c.v. STZ (Bao et al., 2017), and thus, female subjects might not be suitable as the STZ model of AD.

#### 2.3.3 mCRP model of AD

Monomeric C-reactive protein (mCRP) is a proinflammatory molecule yielded by activation and disaggregation of the pentameric CRP. Clinically, it has been associated with the triggering and progression of AD through its deposition in stroke-damaged areas (Slevin et al., 2015, 2020). A recently developed mouse model of dementia by intrahippocampal injection of mCRP may be a new tool to study AD and neuroprotective agents (García-Lara et al., 2021). C57BL/6 J mice injected bilaterally with a dose of 3.5 μg of mCRP show a long-lasting loss of learning and memory. Remarkably, cognitive loss was protected by the oral administration of an anti-inflammatory drug (García-Lara et al., 2021). The mCRP dementia model shows a marked increase in p-tau levels in the hippocampus area (Slevin et al., 2015; García-Lara et al., 2021), whereas a slight increase in Aβ was detected by immunostaining (Slevin et al., 2015). Sex differences have not yet been analyzed in this new model.

#### 2.3.4 Other AD models of neuroinflammation

The rodent models generated with the marine toxin okadaic acid and the neurotoxic metal aluminum are less characterized regarding neuroinflammation. Okadaic acid inhibits selective protein phosphatases. It would induce neuroinflammation and associated memory impairment after i.c.v. injection, but it is mainly used as a model of tau hyperphosphorylation in rats and mice (Nazem et al., 2015). No clear sex differences in susceptibility to okadaic acid have been reported.

Furthermore, administration of aluminum chloride in rats induces brain damage and cognitive impairment, but the underlying mechanisms are not clarified and may involve activation of tau phosphorylation enzymes (Sanajou et al., 2023). Despite suggestions of gender bias in the effect of aluminum in humans, no sex differences have been reported in rodent models.

Finally, neuroinflammation induced by a high-fat diet (HFD) in rodents is also a model of AD neurodegeneration, where neuroinflammation and oxidative stress trigger tau and amyloid pathology and cause cognitive impairment (Sharma, 2021). Several studies have analyzed the effects of antiinflammatory treatments against HFD (Zameer et al., 2020; Sarroca et al., 2021). HFD induces higher neuroinflammation and metabolic impairment in male subjects than female subjects, and this dimorphism likely causes a sex bias as a model of AD (see Sharma, 2021).

#### 2.3.5 Neuroinflammation models of PD

Neuroinflammation plays a role in PD although it may be less decisive for the neurodegeneration triggering and development than in AD. Studies in animal models suggest that neuroinflammation exacerbates the outcome of neurotoxicity induced by specific dopaminergic toxins (Ramsey and Tansey, 2014). Animal models based on the combination of central and peripheral inflammatory challenges show microglial activation and death of dopaminergic neurons after treatment with proinflammatory agents, such as LPS, paired with neurotoxic agents as paraquat and rotenone (García-Revilla et al., 2022).

## 3 Discussion

Most of the experimental rodent models of neurotoxicity-induced neurodegeneration reviewed here have been widely used for pharmacological screening or for studying specific mechanisms of neurodegeneration. They were developed following human findings such as the neurotoxicity of MPTP or rotenone, the amnesia induced by scopolamine, and mental dysfunction associated with sickness behavior by LPS from bacterial infection. Despite its expected translational profile, no model to date reproduces all the features of the corresponding human neurodegenerative disease. We must keep in mind that the crucial factors in the etiology and physiopathology of diseases such as PD and AD are still unclear. On the other hand, rodent models bearing mutations of familiar cases of neurodegenerative disease have gained a lot of prominence in recent years, but they cannot reproduce the whole pathology either. In addition, the bulk of neurodegenerative human cases are age-related, with lengthy processes and not driven by the inheritance of autosomal dominant mutations.

Over the years, considerable progress has been made in understanding PD mechanisms, developing experimental models and testing potential therapeutics. We consider here the latest developments, challenges, and gaps in neurodegeneration models of PD. Animal models have been the primary tool for studying PD due to their genetic and neuroanatomical similarity to humans. Rodent models, such as MPTP and 6-OHDA models, have provided valuable insights into disease pathogenesis. However, their limitations include inadequate modelling of non-motor signs and the lack of α-synuclein aggregation, a hallmark of PD, and the limitation of the MPTP model to a specific mouse strain, as described above. The most recent and advantageous model to date for drug development in sporadic PD is the rotenone model, which can be established in most rodent species and produces α-synuclein aggregates, in the form of Lewy body-like aggregates, in addition to motor and non-motor signs. Nonetheless, the dosage range is critical and near to general toxicity in contrast with the former models that act directly on the CNS. NHP models, particularly macaques, are still instrumental in investigating PD motor symptoms and α-synuclein pathology. Nevertheless, ethical concerns, high costs, and limited availability hinder the widespread utilization of this model.

There exist numerous challenges in the development of improved PD animal models. Among them is the variability in clinical phenotypes: PD exhibits substantial heterogeneity in its clinical presentation, disease progression, sex differences, and response to treatments. Reproducing this diversity in animal models remains a significant challenge. Another challenge is the lack of comprehensive non-motor phenotyping, as non-motor signs, including cognitive impairment and psychiatric disturbances, significantly impact patients’ quality of life. However, these aspects are often overlooked in animal models, limiting our understanding of PD full spectrum. Future developed models should mimic α-synuclein pathology, a major shortcoming in MPTP and 6-OHDA rodent models. Furthermore, there are several lacunae in the current research with PD models. An important omission is the limited longitudinal studies. These studies are essential for understanding disease progression and evaluating potential therapies. Similarly, long-term animal models could offer insights into the chronic nature of PD and its interaction with age-related changes and genetic background. PD is a multifactorial disorder with both genetic and environmental contributions. Current models often focus on either genetic or toxin-induced approaches, neglecting the intricate interplay between genetics and environmental factors.

Animal models of AD based on the wild-type mouse and rat cannot show the overt amyloid pathology of AD transgenic mice (TgAD) because rodent Aβ is not prone to form fibrillary deposits as that of human or non-human primates and other animals (Ueno et al., 2014). Fibrillary tangles are not generated either. AD models of cholinergic dysfunction and neuroinflammation do not recapitulate the proteinopathy linked to the human disorder although they may induce some imbalances in the synthesis or processing of APP and tau, as described above. The absence of these pathological hallmarks of AD is a major limitation of these models because they do not mimic other underlying mechanisms in AD caused by either Aβ aggregation or other factors (Iliyasu et al., 2023; Ratan et al., 2023). However, they model neurodegenerative mechanisms of broad implication in AD and other neurodegenerative diseases. For instance, scopolamine is also used to model cholinergic dysfunction in PD and Lewy body dementia (Zhang et al., 2019) although its main application is in AD. The scopolamine model is useful as a first step for testing compounds against learning and memory loss in AD. Although there is more interest in the development of disease-modifying anti-amyloid or anti-tau drugs, as noted above, cholinesterase inhibitors are still considered valid drugs in the current scenario of AD treatments (Molchan and Fugh-Berman, 2023). The feasibility of peripherally injected scopolamine in mice to produce cognitive loss that can be rescued with cholinergic drugs makes it an advantageous model in many laboratories. However, it is recommended that TgAD mice be used as a next step to more fully characterize the anti-AD mechanisms of selected drugs (Sampietro et al., 2022). Similarly, peripheral injection of the proinflammatory endotoxin LPS in mice is a convenient and useful model for drug development and research in AD and other dementia cursing with neuroinflammation (Nava Catorce and Gevorkian, 2016). However, selected AD anti-inflammatory drugs would also require further testing in a more reliable background such as TgAD mice. STZ has an additional limitation in the requirement of its i.c.v. administration for modeling an AD mouse (Chen et al., 2013). mCRP also requires intracerebral injection, but this model is suitable for a second round of anti-inflammatory drug testing or specific studies of AD dementia associated with cerebrovascular damage. Okadaic acid is also limited by its i.c.v. administration. Aluminum chloride is poorly characterized as the AD model. HFD neuroinflammation is induced by metabolic disorders associated with AD risk, with the consequent limitation for comprehensive testing of protective drugs.

The first challenge is to develop assays of LPS, STZ, and other neurotoxic agents in mice with a humanized APP gene that would yield Aβ prone to form amyloid plaques. These engineered mice have been an important breakthrough in the recent development of the second and third generation of TgAD mice, namely, mice with humanized APP gene bearing either familial AD mutations in this gene or in both APP and PS1 genes, respectively (Sasaguri et al., 2022). There is also a need for standardized protocols of treatment and phenotype characterization, including translational non-invasive biomarkers. The main gaps in the reviewed AD models here are the consideration of the response to the neurotoxic agent with the age and sex of the animals and the agent interaction with alleles of genetic risk. Another gap is the concomitant study of systemic comorbidities.

In addition to their possible utility for pharmacological testing as discussed above for each model, wild-type animal models of neurotoxicity-induced neurodegeneration may contribute to uncovering the role of environmental neurotoxic agents as etiological factors in neurodegenerative diseases.

While there was no enough improvement in finding suitable drugs and therapies against neurodegenerative disease, we should take advantage of all available tools (Drummond and Wisniewski, 2017; Khan et al., 2023). Non-transgenic and transgenic rodents and other animals can be used for specific purposes. Animal experimentation cannot be avoided in drug development in the near future. Where convenient, there is also a range of CNS cell culture models for *in vitro* assays. Furthermore, new advances such as cultures from human-induced pluripotent stem cells obtained from patients, brain organoids, or computational brain modelling will hopefully contribute to therapies that lead to maintaining brain health into old age.

## Author contributions

ER-F contributed to conception and design of the review. CSa, CSu, and ER-F wrote sections of the first draft of the manuscript. CB searched for and organized the references and drew the figures. All authors contributed to manuscript revision, read, and approved the submitted version.
